# White matter free water mediates the associations between placental growth factor, white matter hyperintensities, and cognitive status

**DOI:** 10.1002/alz.14408

**Published:** 2024-12-18

**Authors:** Kyle C. Kern, Manu Vohra, Marissa L. Thirion, Danny J. J. Wang, Donna M. Wilcock, Jeffrey F. Thompson, Gary A. Rosenberg, Abhay Sagare, Abhay Moghekar, Hanzhang Lu, Tiffany Lee, Fanny M. Elahi, Claudia L. Satizabal, Russell Tracy, Sudha Seshadri, Kristin Schwab, Karl Helmer, Herpreet Singh, Pia Kivisäkk, Steven M. Greenberg, Keith Vossel, Joel H. Kramer, Pauline Maillard, Charles S. DeCarli, Jason D. Hinman

**Affiliations:** ^1^ Department of Neurology University of California Los Angeles Los Angeles California USA; ^2^ Department of Neurology West Los Angeles Veterans Affairs Medical Center Los Angeles California USA; ^3^ Departments of Neurology and Radiology University of Southern California, SHN Los Angeles California USA; ^4^ Sanders‐Brown Center on Aging Department of Physiology University of Kentucky Lexington Kentucky USA; ^5^ Center for Memory and Aging Department of Neurology University of New Mexico Albuquerque New Mexico USA; ^6^ Zilkha Neurogenetic Institute Department of Physiology and Neuroscience Keck School of Medicine University of Southern California Los Angeles California USA; ^7^ Department of Radiology Johns Hopkins University Baltimore Maryland USA; ^8^ Department of Neurology Icahn School of Medicine at Mount Sinai New York New York USA; ^9^ Glenn Biggs Institute for Alzheimer's & Neurodegenerative Diseases Department of Population Health Sciences UT Health San Antonio San Antonio Texas USA; ^10^ Departments of Biochemistry and Pathology & Laboratory Medicine Larner College of Medicine University of Vermont Burlington Vermont USA; ^11^ Department of Neurology Massachusetts General Hospital Harvard University Boston Massachusetts USA; ^12^ Memory and Aging Center Weill Institute for Neuroscience University of California San Francisco San Francisco California USA; ^13^ Department of Neurology University of California Davis Davis California USA

**Keywords:** dementia, diffusion tensor imaging, magnetic resonance imaging, placental growth factor, vascular cognitive impairment, white matter hyperintensities

## Abstract

**INTRODUCTION:**

Placental growth factor (PlGF) may regulate cerebrovascular permeability. We hypothesized that white matter interstitial fluid accumulation, estimated via magnetic resonance imaging (MRI) free water (FW), would explain the associations between elevated PlGF, white matter hyperintensities (WMH), and cognitive impairment.

**METHODS:**

MarkVCID consortium participants ≥55 years old with plasma PlGF and brain MRI were included. We tested cross‐sectionally whether FW mediated the associations between PlGF and WMH, or PlGF and cognition, measured using the Clinical Dementia Rating (CDR) scale and an executive function (EF) composite (Uniform Data Set version 3 [UDS3]‐EF).

**RESULTS:**

For 370 participants (mean age 72), a higher PlGF was associated with higher FW, higher WMH, and higher CDR, but not UDS3‐EF. Higher FW was associated with higher WMH, higher CDR, and lower UDS3‐EF. FW explained 26% of the association between PlGF and CDR and 73% of the association between PlGF and WMH.

**DISCUSSION:**

Elevated PlGF may contribute to WMH and cognitive impairment through white matter FW accumulation.

**CLINICAL TRIAL REGISTRATION:**

NCT06284213

**Highlights:**

- PlGF is a promising blood‐based biomarker for vascular cognitive impairment.
- In MarkVCID, higher PlGF was associated with accumulated white matter FW on MRI.
- FW mediated the association between higher PlGF and MRI‐visible white matter injury.
- FW mediated the association between PlGF and worse CDR scale.
- PlGF may contribute to cognitive dysfunction via accumulated interstitial fluid.

## 1 BACKGROUND

Converging evidence supports brain endothelial cell dysfunction as a key driver for the pathogenesis of cerebral small vessel disease (CSVD),^1^ an important contributor to cognitive decline and dementia. However, CSVD is typically diagnosed by magnetic resonance imaging (MRI) markers of downstream white matter injury such as confluent T2 white matter hyperintensities (WMH) of presumed vascular origin. The identification of upstream blood and imaging biomarkers is important for elucidating the pathophysiology of CSVD‐related white matter injury, identifying patients at risk, and monitoring potential treatments for vascular contributions to cognitive impairment and dementia (VCID).

Placental growth factor (PlGF), measured in plasma, has recently been shown to be related to CSVD, WMH, and VCID.^2^ PlGF is a member of the vascular endothelial‐derived growth factors (VEGFs) and signals through the Flt‐1 receptor.^3^, ^4^ In preclinical studies, PlGF signaling is induced by vascular shear stress^5^ and ischemic conditions.^4^ PlGF potentiates the effects of VEGF, contributing to pathophysiological neoangiogenesis and increasing vascular permeability.^6^, ^7^ As a manifestation of endothelial dysfunction, increased blood–brain barrier (BBB) permeability is a key component for the hypothesized progression of CSVD and WMH, leading to extravasation of fluid and inflammatory molecules into the brain parenchyma, subsequent white matter injury, and cognitive decline.^8^, ^9^ Given its association with WMH and cognitive status and the plausible physiologic connection, plasma PlGF might serve as an early stage, cost‐effective biomarker for identifying patients at risk for VCID.

On MRI, CSVD is characterized by the accumulation of WMH, lacunes, and cerebral microbleeds.^10^ WMH increase in prevalence in the fifth decade of life,^11^ but their total volume and accumulation are associated with the risk of stroke, dementia, and death.^12^ Although some data suggest WMH can regress,^13^ they largely reflect late‐stage white matter injury. Diffusion tensor imaging (DTI), particularly DTI‐FW, may detect earlier stages of white matter injury. For example, FW quantifies increased fluid content in brain white matter and is altered even in regions that appear normal on conventional MRI,^14^, ^15^ preceding the progression of new WMH.^16^, ^17^ White matter FW has also been shown to mediate the relationship between elevated systolic blood pressure, arterial stiffness, and WMH burden.^18^ However, the relationship between FW and plausible upstream blood biomarkers of endothelial dysfunction such as PlGF has not been determined.

In proposed models of CSVD, WMH progression, and VCID, endothelial dysfunction and increased vascular permeability lead to white matter injury, contributing to cognitive decline and dementia.^1^, ^19^ As a circulating angiogenic marker with the potential to regulate brain vascular permeability, PlGF may serve as an upstream marker for VCID. Therefore, using data from the MarkVCID consortium, which includes older participants recruited from the community and clinic with a range of vascular risk factors and risk for VCID, we hypothesized that elevated levels of PlGF would be associated with increased FW in white matter. Furthermore, we tested whether increased FW mediated the associations between PlGF and cognition or PlGF and WMH volume.

## 2 METHODS

### 2.1 Participants

MarkVCID is a multisite consortium established to validate candidate biomarkers for CSVD through the recruitment of participants from diverse racial and ethnic backgrounds, with a range of vascular risk factors, and across the spectrum of cognitive impairment (cognitively intact, mild cognitive impairment, or mild dementia). Six MarkVCID sites have their own specific inclusion and exclusion criteria for prospective enrollment of clinic and community volunteers, which were previously described.^20^ All participants provided written, informed consent. All participating site protocols, including the template MarkVCID consent language, were approved by site Institutional Review Boards. All sites used harmonized acquisition and analysis protocols to collect a plasma endothelial signaling biomarker kit^2^ and an imaging‐based biomarker kit.^21^ This cross‐sectional study included data from MarkVCID participants who were at least 55 years old and had PlGF and FW measurements that passed quality control.

### 2.2 Cognitive assessment

Participants were assessed with the Clinical Dementia Rating (CDR) scale^22^ and the Uniform Data Set version 3 (UDS3)^23^ at each site by centrally trained raters. For both the CDR global score (CDR‐gs) and the sum of boxes (CDR‐sb), higher scores indicate worse cognitive status. The UDS3‐EF^24^ is a composite executive function (EF) measure designed for the National Alzheimer's Coordinating Center UDS3. The scale was derived from over 3500 normal control participants (CDR = 0) from 29 Alzheimer's Disease Research Centers. The donor scales were Digit Span Backwards (total correct), Trail Making Test (TMT) parts A and B (correct lines per minute), lexical fluency (F and L words – total correct), and semantic fluency (animal and vegetable fluency – total correct). Item response theory methods were employed to create the overall score. Detailed model‐building steps and validation data are available in Staffaroni et al. UDS3‐EF is sensitive to early changes across the neurodegenerative disease spectrum, and this composite score was previously shown to perform better than any individual component.^24^ Higher UDS3‐EF indicates better performance. While EF has been consistently reported to be associated with cerebrovascular disease,^25^ both CDR and UDS3‐EF were previously used as outcome measures for MarkVCID biomarker validation studies, including PlGF and FW.^2^, ^21^ For this reason, these cognitive assessments were selected a priori for this study.

**RESEARCH IN CONTEXT**

1. **Systematic review**: The authors reviewed online databases for publications and meeting abstracts related to PlGF, cerebrovascular imaging markers, and cognition. In preclinical studies, PlGF helped regulate cerebrovascular permeability. In clinical studies, PlGF has been shown to be related to WMH and cognitive status. Understanding the mechanisms by which PlGF relates to clinical cognitive dysfunction will improve biomarker development and contribute to future treatments.
2. **Interpretation**: Our findings support a proposed role for PlGF in vascular‐mediated white matter injury and cognitive impairment through accumulated white matter interstitial fluid, measured using MRI FW.
3. **Future directions**: Ongoing longitudinal studies will elucidate the temporal dynamics of increased PlGF, FW accumulation, white matter injury, and cognitive decline. Incorporation of BBB permeability imaging techniques would further support the proposed mechanism by which increased PlGF contributes to the progression of vascular‐mediated white matter injury and cognitive decline.

### 2.3 Imaging

Across the MarkVCID sites, three 3T MRI scanner models were used: Siemens TIM Trio, Siemens Prisma, and Philips Achieva. A uniform MRI protocol has been published that was harmonized across sites.^26^ The protocol included (1) a sagittal, 3D, T1‐weighted, multi‐echo magnetization‐prepared rapid acquisition of gradient echo (ME‐MPRAGE) with 1 mm^3^ isotropic resolution; (2) a sagittal, 3D, fluid‐attenuated inversion recovery (FLAIR) with 1 mm^3^ isotropic resolution; and (3) an axial DTI sequence with 2 mm^3^ isotropic resolution, forty directional diffusion images with *b* = 1000 s/mm^2^ and six non‐directional images with *b* = 0 s/mm^2^. A second, reverse polarity DTI with at least one *b* = 0 s/mm^2^ and one *b* = 1000 s/mm^2^ volume was acquired to correct susceptibility distortions.

### 2.4 MRI processing

**FIGURE 1:**
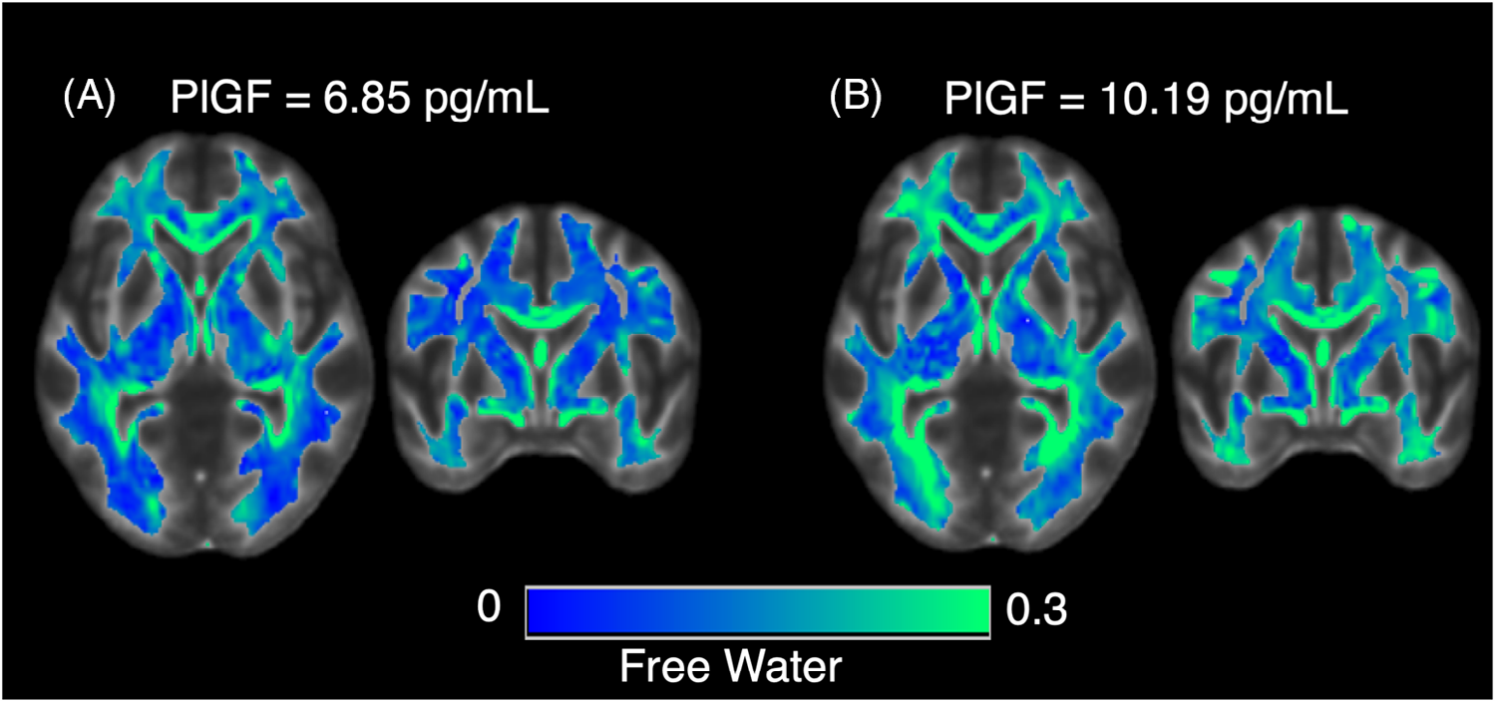
Representative examples of FW images. (A) A participant with a relatively low PlGF (PlGF = 6.85 pg/mL) and mean white matter FWr fraction (FW) of 0.16. (B) An age‐ and sex‐matched participant with relatively high PlGF (10.19 pg/mL) and FW of 0.22. FW, free water; PlGF, placental growth factor.

DTI images were used to calculate the mean white matter FW fraction using the MarkVCID FW biomarker kit. Instrumental and clinical validation of the FW biomarker kit has been published,^21^ and the scripts are publicly available (markvcid.partners.org). In brief, DTI was corrected for head motion and eddy current distortions, and a tensor was fit at each voxel. Conventional fractional anisotropy (FA) images were derived from the tensor, while FW images were calculated by fitting a two‐compartment model to the single‐shell diffusion data.^27^ FW was modeled as an isotropic, fast‐diffusing compartment with diffusion characteristics similar to cerebrospinal fluid (CSF), while the remaining diffusion signal comprised an anisotropic, slower‐diffusing tissue compartment. The FW fraction is unitless and measures the fraction of the diffusion signal attributable to unhindered water molecules in the extracellular space, ranging from 0 to 1 (Figure 1). FA and FW images were non‐linearly aligned to a FA template where a white matter mask was used to calculate the mean FW across the white matter for each participant.

WMH volumes were calculated using a previously validated MarkVCID WMH biomarker kit using 3D FLAIR and T1 images.^21^ In brief, the T1 image was used to remove non‐brain material, segment tissue into gray matter, white matter, and CSF, and non‐linearly align to a minimal deformation template. The FLAIR image was intensity‐normalized, aligned to the T1, and then spatially transformed to the template. A Bayesian segmentation algorithm then resegmented tissue into WMH, gray matter, white matter, and CSF using T1 and FLAIR images in template space. Tissue segmentations were reverse transformed into native T1 space and used to calculate tissue volumes. WMH volumes were converted to a percentage of the total intracranial volume (gray matter + white matter + CSF) to adjust for head size and then log‐transformed (logWMH) to account for a skewed distribution.

### 2.5 Plasma biomarker measurements

Plasma PlGF levels were measured as previously described using the MarkVCID plasma endothelial signaling kit,^2^ which was previously shown to have excellent test–retest and across‐site reliability. In brief, at least 5 mL plasma was collected in 10‐mL EDTA vacutainers, gently inverted 5 to 10 times, and then centrifuged at 2000 × *g* for 10 min. Plasma was aliquoted and frozen at −80°C until analysis. PlGF was measured in technical duplicate using the MesoScale Discovery V‐PLEX Angiogenesis Panel 1 Human Kit (K15190D; Limit of Quantitation (LOQ) = 1.50 to 800 pg/mL). The mean coefficient of variation (CoV) across all participants was 3.8%. Cystatin‐C was also measured as a marker of renal clearance in 90% of participants. Plasma samples were used to measure cystatin C by U‐PLEX Human Cystatin C Assay (Mesoscale Discovery No. K151MK9; LOQ = 23.9 to 23,000 pg/mL) according to the manufacturer's protocol by two labs (University of California, Los Angeles and University of Texas Health Science Center at San Antonio).

### 2.6 Statistical analysis

Outcomes included CDR‐gs, UDS3‐EF, and logWMH. For one participant UDS3 was not completed, and for one participant WMH volume could not be calculated. We used multivariable regression while covarying for age and sex to determine whether PlGF was associated with FW and whether PlGF or FW was associated with logWMH. For cognitive outcomes, covariates included age, sex, and education. For seven participants, educational attainment was not recorded, so the missing covariate data were single‐imputed from a linear regression model of the complete case data using age, sex, and study site to estimate educational attainment. Multivariable regression was used to separately test whether PlGF or FW was associated with UDS3‐EF, and ordinal logistic regression was used to test for associations with CDR‐gs. Standard mediation path analysis^28^ was used to test whether FW mediated the association between PlGF and CDR‐gs or the association between PlGF and WMH burden. Structural equation modeling (for logWMH) or generalized structural equation modeling (for CDR‐gs) was used to test each path of the mediation analysis: independent predictor to mediator (a), mediator to dependent outcome (b), independent predictor to dependent outcome total effect (c), direct effect (c’), and indirect (mediated) effect. Since cystatin‐C was available for 90% of participants (*n* = 331), we repeated the foregoing analyses including cystatin‐C as an additional covariate to test whether renal clearance moderated the associations with PlGF. As an additional sensitivity analysis, since the CDR‐gs can be biased toward memory impairment, we also tested for associations and mediation using CDR‐sb. Linear regression and mediation results are reported as standardized beta coefficients (β), while ordinal logistics regression results are reported as adjusted odds ratio (aOR). Analyses were performed using Stata version 17 (stata.com).

## 3 RESULTS

### 3.1 Demographics

The study included 370 participants from six sites. The mean age (± SD) was 72 ± 8 years, and 61% were female. Most participants identified as White (83%), 47% as Hispanic ethnicity. As measured by the CDR‐gs, 60% were cognitively unimpaired. CDR‐sb ranged from 0 to 6, with a median of 0 and an interquartile range of 1. On MRI, 57% had a severe WMH burden (Fazekas grade 3). Table 1 includes participant characteristics.

**TABLE 1:** Participant characteristics.

*N*	370
Age in years (SD^a^)	72 (8)
Percentage female (*n*)	61% (227)
Race (*n*)	
White	83% (306)
Black	5.9% (22)
American Indian/Alaska Native	1% (4)
Asian	<1% (2)
Hispanic ethnicity (*n*)	47% (173)
Study site (*n*)	
Johns Hopkins University	12% (43)
UCSF	9% (34)
University of Kentucky	25% (95)
University of New Mexico	9% (32)
USC	16% (61)
UT San Antonio	28% (105)
Risk factors (*n*)	
Hypertension	52% (192)
Hyperlipidemia	51% (190)
Diabetes	20% (70)
Cardiovascular disease	7% (25)
Atrial fibrillation	3% (12)
Congestive heart failure	2% (6)
Prior stroke	7% (25)
Years of education (median, IQR^b^)	16 (5)
CDR^c^ global score (*n*)	
0	60% (221)
0.5	38% (139)
1	3% (10)
CDR sum of boxes (median, IQR)	0 (1)
UDS3^d^ executive function (median, IQR)	−0.49 (1.20)
Fazekas	
0	4% (13)
1	19% (71)
2	20% (74)
3	57% (212)
WMH^e^ volume (mL, median, IQR)	2.86 (6.44)
FW fraction (median, IQR)	0.22 (0.059)
PLGF^f^ in pg/mL (median, IQR)	7.80 (3.67)
Cystatin‐C in pg/mL (median, IQR)	1971284 (1083957)

^a^ Standard deviation,

^b^Interquartile range,

^c^Clinical Dementia Rating scale,

^d^Uniform Dataset version 3

^e^White matter hyperintensity,

^f^Placental growth factor.

### 3.2 Regression associations

#### 3.2.1 PlGF

**FIGURE 2:**
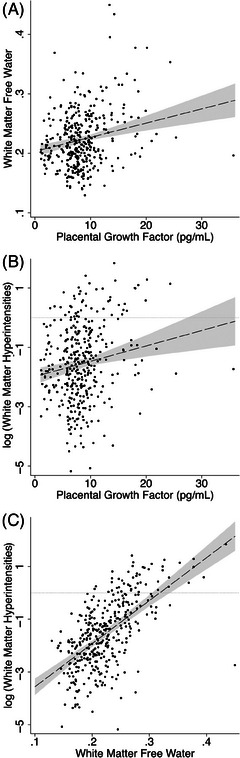
PlGF is associated with imaging markers of white matter injury. (A) Higher PlGF was associated with higher white matter FW (*n* = 370, standardized beta (β) 0.21, 95% CI: 0.12 to 0.30, *p* < .001). (B) Higher PlGF was also associated with greater WMH volume, converted to a percentage of intracranial volume and log‐transformed (logWMH) (*n* = 369, β = 0.17, 95% CI: 0.073 to 0.26, *p* = .001). C) Higher FW was associated with higher logWMH (*n* = 369, β = 0.60, 95% CI: 0.51 to 0.69, *p* < .001). Dashed lines and gray areas show predicted values with 95% confidence intervals while covarying for age and sex. FW, free water; PlGF, placental growth factor; WMH, white matter hyperintensity.

**FIGURE 3:**
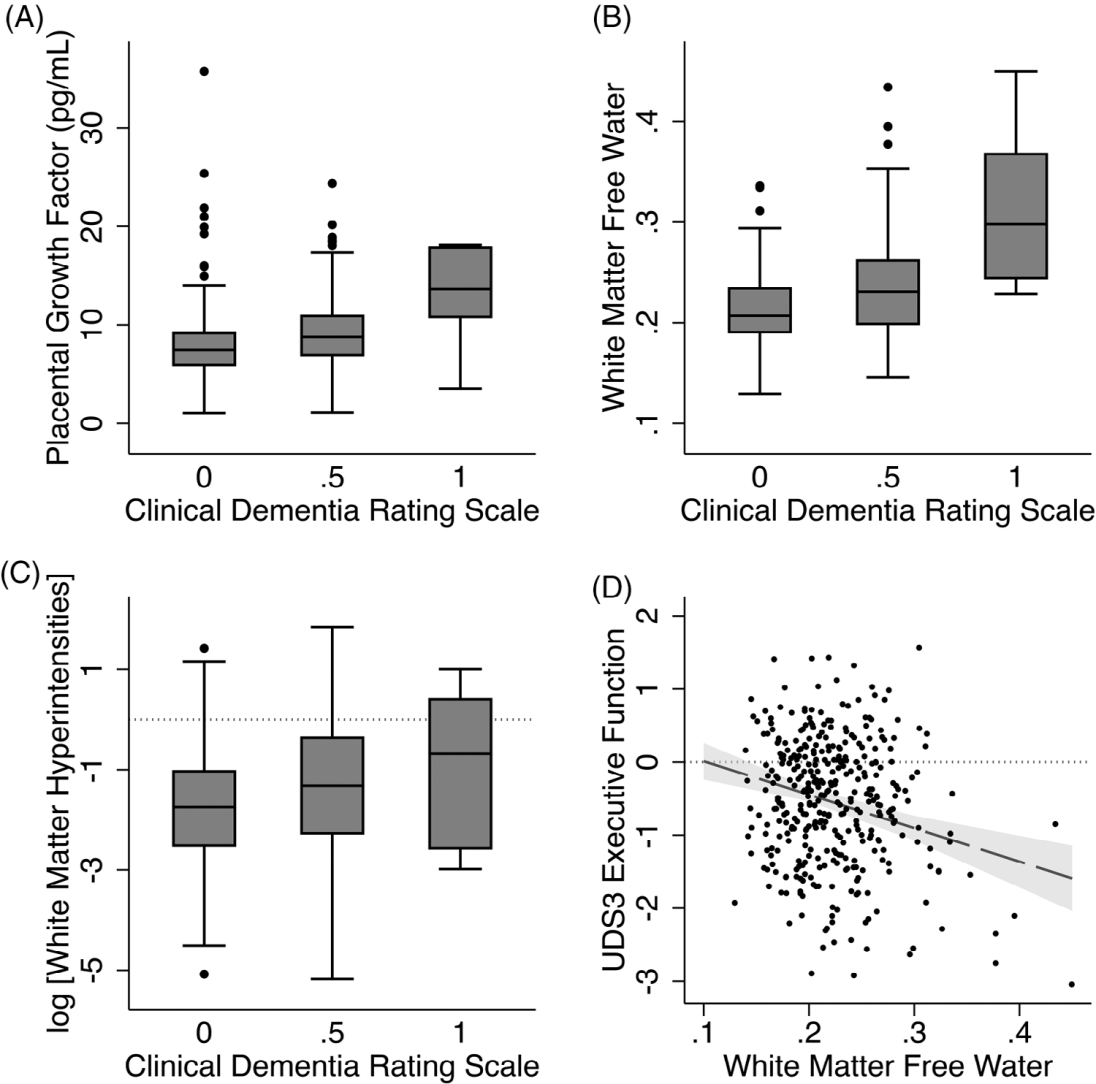
PlGF and white matter FW are each associated with cognition. (A) The box plot shows that higher PlGF was associated with higher CDR‐gs (*n* = 370, adjusted OR 1.67 per standard deviation increase in PlGF, 95% CI: 1.31 to 2.13, *p* < .001). (B) Box plot shows that higher FW was also associated with higher CDR‐gs (*n* = 370, OR 2.13 per standard deviation increase in FW; 95% CI: 1.64 to 2.75; *p* < .001). (C) Box plot shows that greater log‐transformed WMH volume (logWMH) was also associated with higher CDR (*n* = 369, OR 1.48 per standard deviation increase in logWMH; 95% CI: 1.17 to 1.87; *p* = .001) (D) Only higher FW was associated with higher score on UDS3‐EF (*n* = 369, standardized β = −0.25, 95% CI: −0.36 to −0.14, *p* < .001). Dashed line and gray area show predicted values with confidence intervals while covarying for age, sex, and educational attainment. CDR‐gs, Clinical Dementia Rating scale global score; FW, free water; OR, odds ratio; PlGF, placental growth factor; UDS3‐EF, Uniform Data Set 3 executive function composite; WMH, white matter hyperintensity.

Higher PlGF was associated with higher FW (β: 0.21, 95% CI: 0.12 to 0.30, *p* < .001; Figure 2A) and higher logWMH (β: 0.17, 95% CI: 0.073 to 0.26, *p* = .001; Figure 2B). Higher PlGF was also associated with higher CDR‐gs (aOR: 1.67 per standard deviation increase in PlGF, 95% CI: 1.31 to 2.13, *p* < .001; Figure 3A), but not the UDS3‐EF score.

#### 3.2.2 FW

Higher white matter FW was associated with higher logWMH (β: 0.60, 95% CI: 0.51 to 0.69, *p* < .001; Figure 2C) and was also associated with higher CDR‐gs (aOR 2.13 per standard deviation increase in FW; 95% CI: 1.64 to 2.75; *p* < .001; Figure 3B) and lower UDS3‐EF score (β: −0.25; 95% CI: −0.36 to −0.14; *p* < .001; Figure 3D).

#### 3.2.3 WMH

Higher logWMH was also associated with higher CDR‐gs (aOR: 1.48 per standard deviation increase in logWMH; 95% CI: 1.17 to 1.87; *p* = .001; Figure 3C) but was not significantly associated with UDS3‐EF score (β: −0.092; 95% CI: −0.19 to 0.010; *p* = .077).

### 3.3 Mediation analyses

#### 3.3.1 Mediation of cognitive status

**FIGURE 4:**
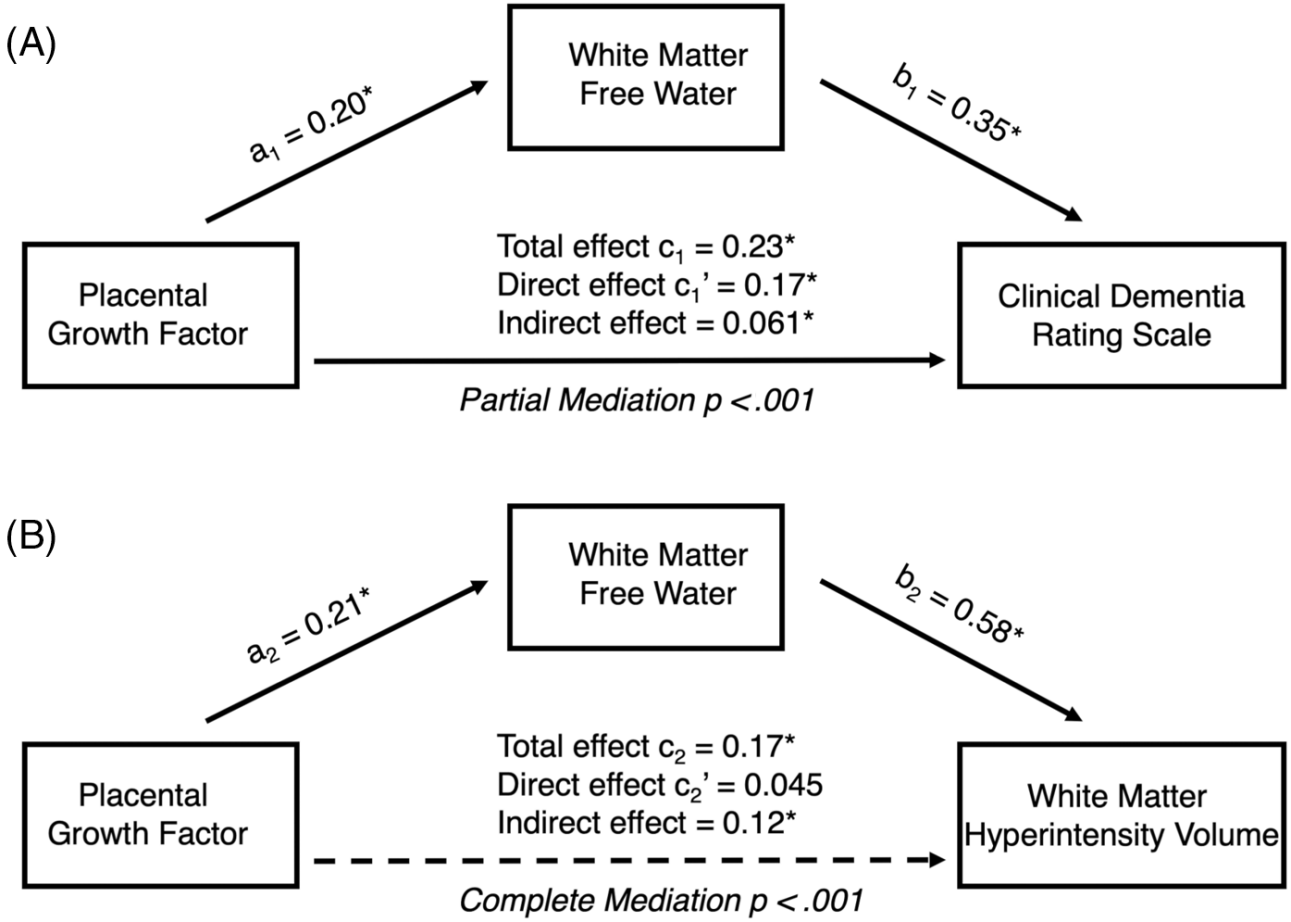
FW mediates association between PlGF and cognitive status. Structural equation modeling was used to determine the mediating effect of white matter FW on associations between PlGF and either CDR‐gs or WMH volume (logWMH). Each path in the mediation analysis is reported as a standardized beta coefficient. (A) While there was an association between PlGF and CDR‐gs (c1: *p* < .001) when FW was included as a mediator, the direct effect (c1’) was reduced compared to the total effect (c1) but was still significant (*p* = .001), signifying partial mediation through FW (indirect effect: *p* = .001). FW explained 26% of the association between PlGF and CDR‐gs while also covaring for age, sex, and education. (B) There was also an association between PlGF and logWMH (c2: *p* = 0.001) that was no longer significant (c2’: *p* = 0.25) when FW was included as a mediator. Thus, FW fully mediated the relationship between PlGF and logWMH, explaining 73% of the association (*p* < .001) while covarying for age and sex. *p ≤  .001. CDR‐gs, Clinical Dementia Rating scale global score; FW, free water; PlGF, placental growth factor; WMH, white matter hyperintensity.

Since our primary goal was to test the hypothesis that FW mediated the relationship between PlGF and CDR‐gs, we used structural equation modeling. In a model that included FW as a mediator and age, sex, and education as covariates, the direct effect of PlGF on CDR‐gs was 0.17 (95% CI: 0.07 to 0.27; *p* = .001). The indirect effect of PlGF on CDR‐gs through FW as a mediator was 0.061 (95% CI: 0.026 to 0.096, *p* < .001). Thus, FW partially mediated the relationship between PlGF and CDR‐gs, explaining 26% of the association. The mediation analysis is depicted in Figure 4A. When CDR‐sb was used in place of CDR‐gs, the results remained significant (Supplemental Materials).

#### 3.3.2 Mediation of visible white matter injury

Since PlGF is believed to regulate vascular permeability, and PlGF was previously shown to be associated with WMH, we secondarily tested whether this association was also mediated by white matter FW using structural equation modeling. While there was a significant total effect of PlGF on logWMH as described earlier, when FW was included in the model as a mediator, the direct effect of PlGF on logWMH was no longer significant (0.045, 95% CI: −0.035 to 0.13; *p* = .27). Meanwhile, there was a significant indirect effect of PlGF on logWMH through FW as a mediator of 0.12 (95% CI: 0.067 to 0.18, *p* < .001). These results indicated full mediation, with FW explaining 73% (indirect/total effect) of the association between PlGF and logWMH (Figure 4B).

### 3.4 Cystatin‐C adjustment

Given that differences in renal function could alter plasma biomarker measurements, we performed sensitivity analyses in a subset of 331 participants for which cystatin‐C measures were available. Higher cystatin‐C was associated with increased FW (β: 0.26; 95% CI: 0.16 to 0.35; *p* < .001) and increased logWMH (β: 0.19, 95% CI: 0.091 to 0.29, *p* < .001). But cystatin‐C was not associated with PlGF (β: 0.075: 95% CI: −0.033 to 0.18; *p* = .17) (Figure S1), UDS3‐EF, or CDR‐gs. When cystatin‐C was included as an additional covariate in secondary prior mediation analyses, there were no significant changes to the results.

## 4 DISCUSSION

In this multicohort, cross‐sectional study that included participants with a range of vascular risk factors and cognitive status ranging from unimpaired to mild dementia, we found that increased plasma PlGF was associated with higher white matter FW and that FW partially mediated the association between PlGF and cognitive status, as measured by the CDR global score. As a secondary analysis, we found that FW fully mediated the association between PlGF and WMH volume, an established marker of CSVD. Although this study was cross‐sectional, these results are consistent with a proposed pathophysiological model in which elevated PlGF increases vascular permeability, leading to accumulation of interstitial fluid in the white matter (higher FW), development of WMH, and subsequent cognitive impairment. Longitudinal studies are needed to confirm the proposed temporal dynamics between PlGF, FW, white matter injury, and cognitive decline.

PlGF is a promising blood‐based biomarker for CSVD that we previously showed to be associated with WMH and cognitive status.^2^ In preclinical studies, PlGF potentiates the actions of VEGF and helps to regulate pathophysiologic neocollateralization.^6^, ^7^ PlGF is believed to be upregulated by vessel shear stress^5^ and in ischemic conditions.^4^ PlGF increases vascular permeability, likely through the recruitment of monocytes to arterial walls, thereby promoting vascular inflammation and edema formation.^29^

CSVD is characterized by arteriolosclerosis, stenosis, and reduced compliance of penetrating small arterioles, predominantly in brain regions with the largest blood pressure gradients.^30^, ^31^ These vascular changes predispose vulnerable brain white matter to intermittent hypoperfusion, contributing to endothelial dysfunction, inflammation, and ischemia.^31^ Thus, we hypothesize that PlGF is upregulated in CSVD due to both hypertensive shear stress and intermittent hypoperfusion. The upregulation of PlGF may contribute to increased BBB permeability, which is seen in CSVD and is thought to be pathogenic.^32^ Extravasation of fluid and inflammatory mediators into the white matter interstitium may be a driver of WMH progression and subsequent cognitive decline. Neoangiogenesis, another proposed role for upregulated PlGF, is seen after ischemic stroke^33^ and retinal ischemia^34^ and has been reported in Alzheimer's disease^35^ but is not reported as a histopathologic feature of CSVD. However, CSVD is associated with the elevation of several pro‐angiogenic factors,^36^ and endothelial cell dysfunction and proliferation are evident.^37^

As a biomarker for CSVD or VCID, PlGF could be used as a cost‐effective screening tool for identifying patients at risk for subclinical cerebrovascular disease and insidious cognitive decline. PlGF is already used clinically as a marker for pre‐eclampsia.^38^ For pregnant women, PlGF should be elevated (>100 pg/mL) to promote placental growth, but low PlGF < 12 pg/mL is indicative of high risk for severe pre‐eclampsia and preterm delivery.^39^ For patients at risk of CSVD or VCID, high PlGF > 10 pg/mL has been proposed as a threshold for detecting cognitive impairment (CDR‐gs > 0) with severe WMH (Fazekas > 2).^2^ It remains unknown whether PlGF changes in response to commonly used CSVD risk factor treatments.

Future work should also investigate the modification of PlGF as a potential treatment target to slow CSVD progression. In vitro studies suggest a neurotrophic and neuroprotective effect of PlGF in the setting of oxygen and glucose deprivation.^40^ Furthermore, in a mouse middle cerebral artery occlusion model, mesenchymal stem cells transfected with a viral vector coding for PlGF reduced infarct size and improved motor function outcomes.^41^ Finally, in mouse models of myocardial infarction, administration of recombinant PlGF induced angiogenesis^4^ and improved survival and cardiac function.^42^ In contrast, anti‐PlGF treatment, in the form of monoclonal antibody TB‐403, has been used as an anti‐angiogenic cancer treatment in Phase I clinical studies.^43^

FW is a sensitive marker for white matter changes that occur with aging and CSVD^18^ and is strongly associated with several cognitive measures that relate to VCID,^44^ often outperforming conventional imaging measures.^45^ While elevations in white matter FW fraction likely reflect increased interstitial fluid, the measure cannot be entirely specific to the biological process, since it is estimated by applying a theoretical model to the diffusion MRI signal. DTI data collected using B‐values around 1000 s/mm^2^ largely reflect extracellular diffusion, but they also measure a smaller component of intracellular diffusion and tissue perfusion that may contribute to overestimation of the FW fraction, particularly when applied to single‐shell DTI data.^46^, ^47^, ^48^ However, most clinical studies use single‐shell DTI acquisitions since they are shorter than multi‐shell acquisitions. Across multiple studies, single‐shell FW measures have been strongly associated with CSVD and VCID,^15^, ^18^, ^44^, ^45^, ^49^ supporting its use as a sensitive biomarker for CSVD‐related white matter changes.

While elevated PlGF may contribute to increased vascular permeability and FW estimates tissue fluid content, the link between PlGF and BBB permeability has not been established in clinical studies. Dynamic gadolinium contrast imaging has been used to visualize increased BBB permeability in CSVD,^8^, ^9^ and greater BBB disruption is associated with a higher burden of WMH and longitudinal changes in white matter diffusion metrics.^50^, ^51^ Newer techniques permit mapping of BBB water exchange even without contrast. These arterial spin labeling (ASL) techniques include diffusion‐prepared pseudo‐continuous ASL (DP‐ASL),^52^ multi echo‐time ASL,^53^ and water extraction with phase‐contrast arterial spin tagging (WEPCAST).^54^ In older, non‐demented participants, white matter FW was found to mediate the association between BBB water exchange (using DP‐ASL) and EF.^55^ Thus, future studies should include BBB imaging to test a more complete path analysis that includes BBB permeability as a mediator between PlGF and FW as a driver of cognitive decline.

While FW was associated with cognition using both the CDR and the UDS3‐EF, PlGF was only associated with CDR, and FW only partially mediated the association between PlGF and CDR. Notably, the direct effect of PlGF on CDR was almost three times stronger than the indirect effect. Therefore, although our findings support our proposed model that elevated PlGF contributes to increased vascular permeability, accumulation of interstitial fluid, and subsequent white matter degeneration and cognitive decline, PlGF may also act on CDR through other mechanisms. In this regard, PlGF's potential role in neoangiogenesis, endothelial cell proliferation, and endothelial dysfunction should be further investigated as it relates to both CSVD and Alzheimer's pathology. While hypervascularity is not reported as a pathological feature of CSVD, some preclinical data suggest that amyloid promotes neoangiogenesis,^56^ and hypervascularity is seen with Alzheimer's pathology.^35^ Whether elevated PlGF also relates to cognitive decline through neurodegenerative mechanisms is unknown. Furthermore, CDR is a functional measure dependent on more than just white matter integrity and EF, and we did not assess PlGF's potential contribution to gray matter atrophy. The inclusion of neurodegenerative biomarkers in future studies would help determine the specificity of PlGF for VCID versus other neurodegenerative conditions.

PlGF was not associated with cystatin‐C, and the inclusion of cystatin‐C in regression and mediation models did not alter the results. Thus, it may be reasonable to use PlGF as a biomarker without correcting for renal clearance. On the other hand, we also found associations between cystatin‐C and both imaging markers of white matter injury – FW and WMH. Chronic kidney disease is an established risk factor for CSVD, WMH, and cognitive impairment.^57^, ^58^ Small vessel pathology in the kidney is likely to co‐occur with small vessel pathology in the brain since both organs are susceptible to the effects of chronic hypertension and systemic vascular disease. However, renal dysfunction also contributes to elevated inflammatory or procoagulant factors that exacerbate brain white matter injury. Interestingly, a recent study found increased BBB permeability in participants with end‐stage kidney disease.^59^ Further work is needed to disentangle the effects of kidney dysfunction from systemic vascular disease on brain health.

The strengths of this study include a large sample across multiple sites with a range of vascular risk profiles and cognition ranging from unimpaired to mild dementia. Each biomarker kit was validated for reproducibility across multiple sites and multiple patient cohorts. On the other hand, the study used preliminary data and MRIs from multiple scanners. Furthermore, the cross‐sectional design prevented any temporal or causal conclusions about the relationships between PlGF, FW, WMH, and cognition. However, recruitment is ongoing for MarkVCID2, and longitudinal analyses are planned to better understand how these biomarkers could be used in clinical trials for VCID.

In older participants with a range of vascular profiles at risk of cognitive decline, elevated PlGF relates to white matter injury and cognitive status through increased white matter water content. These associations support a proposed model for how CSVD leads to cognitive impairment, whereby elevated PlGF contributes to increased vascular permeability, leading to the accumulation of white matter injury and cognitive impairment. These results support the use of PlGF and FW as biomarkers for CSVD and VCID treatment trials. PlGF should be tested as a cost‐effective screening tool for identifying patients at risk of vascular‐mediated cognitive impairment.

## CONFLICT OF INTEREST STATEMENT

The authors have no conflicts of interest to disclose.

## CONSENT STATEMENT

All human subjects provided informed consent.

## Supporting information



Supporting Information

Supporting Information

Supporting Information

## Data Availability

The corresponding author had access to all data and took full responsibility for carrying out the research and the integrity of the data, analysis, and interpretation. Interested parties can request MarkVCID data and/or biosamples by following the procedure outlined on the MarkVCID consortium website (markvcid.partners.org) through a formal data request.
