# Supplementary figures and images for: White matter free water mediates the associations between placental growth factor, white matter hyperintensities, and cognitive status
Source: Alzheimers Dement. 2024 Dec 18;21(2):e14408. doi: 10.1002/alz.14408 (PMC11848340; doi:10.1002/alz.14408)

**A**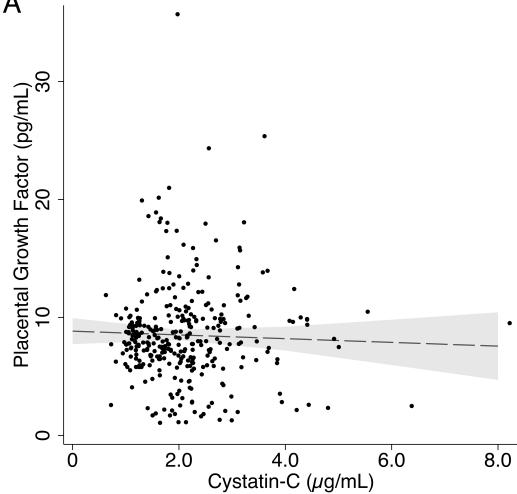**B**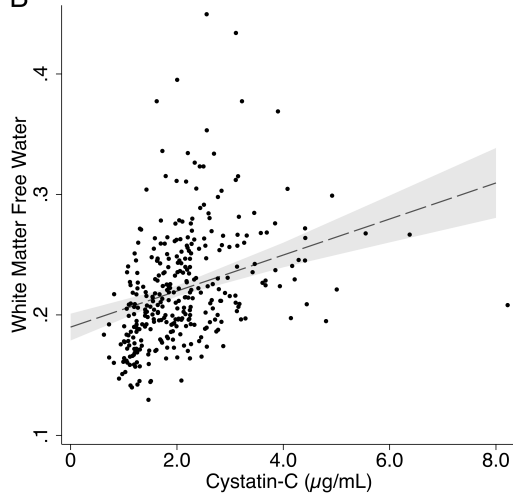**C**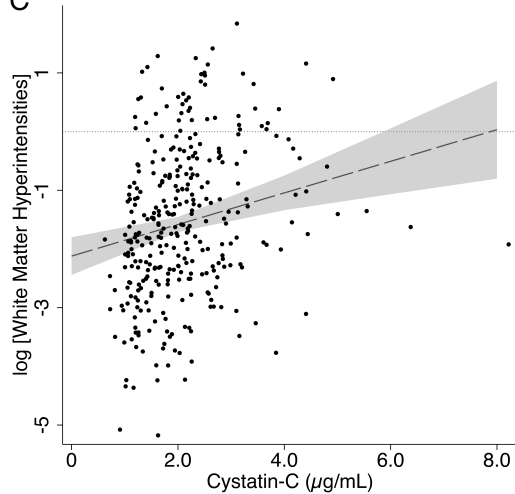

Supplement: Supplementary file 1 — Supporting Information [file ALZ-21-e14408-s003.pdf]
