# Supplementary material for: White matter free water mediates the associations between placental growth factor, white matter hyperintensities, and cognitive status
Source: Alzheimers Dement. 2024 Dec 18;21(2):e14408. doi: 10.1002/alz.14408 (PMC11848340; doi:10.1002/alz.14408)
Supplement: Supplementary file 2 — Supporting Information [file ALZ-21-e14408-s001.docx]

**Supplemental Results**

Clinical Dementia Rating Scale Sum of Boxes

Since Clinical Dementia Rating Scale global score (CDR-gs) may be biased toward memory impairment, as a sensitivity analysis we tested whether the same associations were present with the Clinical Dementia Rating Scale sum of boxes score (CDR-sb). Placental growth factor (PlGF) (adjusted odds-ratio 1.89 per standard deviation, 95% CI: 1.50 to 2.39, p<0.001), mean white matter free water (FW) (aOR 1.84 per standard deviation, 95% CI: 1.47 to 2.31, p<0.001), and log-transformed white matter hyperintensity fraction (logWMH) (aOR 1.40 per standard deviation, 95% CI: 1.12 to 1.74, p=0.003) were each separately associated with CDR-sb while covarying for age, sex, and educational attainment.

In mediation analysis, the direct effect of PlGF on CDR-sb was 0.25 (95% CI 0.16 to 0.34; p<0.001). The indirect effect of PlGF on CDR-sb through FW as a mediator was 0.069 (95% CI: 0.031 to 0.11, p<0.001). FW partially mediated the relationship between PlGF and CDR-sb, explaining 22% of the association, similar to the mediation effect with CDR-gs found in the primary analysis.

**Supplemental Figure**

**Associations with Renal Function**: For 90% of participants who also had plasma cystatin-C measurements: A) cystatin-C was not associated with plasma placental growth factor (n=331, standardized Beta (ß) = 0.075, 95% CI: -0.033 to 0.18, p=0.017). B) However, cystatin-C was associated with white matter free water (n=331, ß = 0.26, 95% CI: 0.16 to 0.35, p<0.001) and C) log-transformed white matter hyperintensity fraction (n= 330, ß = 0.19, 95% CI 0.091 to 0.29, p<0.001). When cystatin-C was included as an additional covariate in secondary mediation analyses, the results remained significant. Dashed line indicates predicted values while covarying for age and sex.
